# Staying in Touch During COVID-19: Long-Term Care Facility Communication Strategies and Family Perceptions

**DOI:** 10.1093/geroni/igab046.606

**Published:** 2021-12-17

**Authors:** Jane Straker, Mi Sun Choi

**Affiliations:** 1 Miami University, Oxford, Ohio, United States; 2 Silla Universiy, Silla University, Pusan-jikhalsi, Republic of Korea

## Abstract

During the COVID-19 pandemic, family concerns regarding residents in long-term care facilities (LTCFs) increased dramatically due to the higher proportion of deaths in LTCFs and an inability to visit, observe care, or easily communicate with residents. However, little is known about how these facilities communicated with families and how communications were related to family perceptions about the facility. To address these knowledge gaps, we implemented an online survey of family members or friends of residents in LTCFs from April 28 to June 19, 2020. A total of 174 responses nationwide reported the types of communications used, frequency of communication and alternative visits, and whether the families had peace of mind, would recommend the facility and whether they were considering removing the resident from the facility. We performed chi-square and t-tests to identify differences in perception among families. Results showed that respondents had more negative perspectives of a facility when they were not informed about confirmed COVID cases. There were no differences in family members’ perceptions of a facility based on the frequency of alternative visits. When respondents could communicate with their family members in an LTCF by telephone, email, mail, and window visits, they had more peace of mind. Respondents were more likely to recommend the facility to others when they were able to communicate via mail with the facility. Our findings suggest multiple communications and transparency about COVID status were most effective in keeping positive family perceptions about the facility. Our results can inform future facility communication protocols.

